# Risk factors for non-cure among new sputum smear positive tuberculosis patients treated in tuberculosis dispensaries in Yunnan, China

**DOI:** 10.1186/1472-6963-11-97

**Published:** 2011-05-11

**Authors:** Hua Jianzhao, Susan van den Hof, Xu Lin, Qiu Yubang, Hou Jinglong, Marieke J van der Werf

**Affiliations:** 1 Yunnan Provincial Centers for Disease Control and Prevention, Yunnan, China; 2 KNCV Tuberculosis Foundation, The Hague, The Netherlands; 3 Center for Infection and Immunity Amsterdam (CINIMA), University of Amsterdam, Amsterdam, The Netherlands

## Abstract

**Background:**

Yunnan province in China has a high tuberculosis (TB) burden. Cure rates in general are high, but they were below the target of 85% in 26 out of 129 counties in 2005. In these 26 counties we assessed which patient-related and treatment-related factors were associated with non-cure.

**Methods:**

We conducted a prospective cohort study. Smear positive pulmonary TB patients treated at the local Center for Disease Control and Prevention (CDC) were interviewed before start of treatment and during the fifth month of treatment using structured questionnaires. Information on treatment outcome was extracted from patient records. Patients cured at the end of treatment were compared to patients with unsuccessful treatment outcomes (failure, default, and death).

**Results:**

A total of 841 patients were registered between January-June 2007 of which 792 (94%) were cured. Independent risk factors for non-cure were having a low income (<3000 RMB per year), not having medical insurance, a delay in health care seeking >30 days, a positive smear test result two months after start of treatment, not being aware of the need to go to the CDC for medical follow up during treatment, and not seeing the need for treatment observation.

**Conclusion:**

Reducing the financial burden of TB disease and providing health education to improve compliance with treatment could increase the proportion of patients with successful treatment outcomes.

## Background

High quality tuberculosis (TB) treatment is a key factor of the Directly Observed Treatment Short-course (DOTS) strategy. In combination with a high case detection rate it is expected to reverse the TB epidemic. The targets for case detection and treatment success are 70% and 85% respectively [1]. Treatment success is defined as bacteriologically confirmed cure or a completed course of treatment without bacteriological proof of cure [2]. TB patients with an unsuccessful treatment outcome, especially failure and default, are a public health concern as these patients may have developed drug resistance and remain infectious for prolonged periods of time.

TB treatment outcomes are influenced by bacterium characteristics such as drug resistance, patient characteristics, patient behavior, and quality of health care [3-8]. Poor adherence to anti-tuberculosis treatment is considered the most important factor leading to non-curein patients infected with strains susceptible to the drugs [9-11]. Therefore, observation of treatment is considered one of the key elements of the DOTS strategy. It has been described before that in some places directly observed treatment (DOT) by village health workers was not very successful, due to insufficient financial incentives for the village health workers in relation to the task of thrice-weekly DOT for 6-8 months [12]. In remote areas, DOT can also be performed by an educated family member or community volunteer [2]. The influence of this approach on treatment outcomes is not known.

Yunnan province in southern China is a high TB burden province with poor economic status. The province covers 394,000 km^2 ^of which 94% is mountainous area. Seventy-four percent of the 44 million inhabitants live in rural areas. Under China's National TB Control Program (NTP), Yunnan province has implemented the DOTS strategy since 2002. In 2004, based on estimates by the Ministry of Health, the provincial case detection rate reached 70% [13]. In 2005, the average treatment success rate was 92% and the average cure rate was 92%. In 24 out of the 129 counties in Yunnan the treatment success rate was below 85% and in 26 counties the cure rate was below 85%.

The Center for Disease Control and Prevention (CDC) of Yunnan province decided to assess which TB patient-related and treatment-related factors are the main determinants for non-cure in the counties of Yunnan province in China with a relatively low cure rate. Therefore, we conducted a prospective cohort study in counties with a cure rate below 85% in 2005. The results may be used to develop policies to improve treatment success in those areas.

## Methods

### Setting, study design, and population

A prospective cohort study was performed in these counties to identify factors that are associated with non-cure. All new sputum smear-positive TB patients over 14 years of age registered between January and June 2007 by the local CDC in the 26 counties with a cure rate <85% in 2005 were eligible for inclusion in our prospective cohort study. Informed consent was taken before inclusion in the study.

### Sample size

For the sample size calculation we considered type of treatment observer as the main factor associated with treatment outcome. We assumed that 67% of the TB patients are observed by a family member and the remaining 33% are observed by a village doctor (ratio 2:1, based on expert opinion). We wanted to be able to show a difference in treatment success rate of 10% between the two groups (85% in those observed by a family member and 95% in those observed by a village doctor). Taking into account a maximum of 15% missing data, a power of 0.8 and alpha of 0.05, a total sample size of 744 patients was required.

### Data collection

All eligible TB patients were approached by the local CDC doctors. TB patients who agreed to participate in the survey were interviewed before start of TB treatment and during the fifth month of treatment using structured questionnaires. In the first interview we collected information about patient characteristics, such as age, sex, educational background, TB symptoms, and co-morbidity (diabetes, HIV/AIDS, lung cancer, liver and kidney diseases, and other co-morbidity). Information on treatment adherence, side effects, patient's knowledge of TB treatment and perceived need for DOT was included in the second interview. At that time, treatment observers were also interviewed to collect information on direct observation of treatment and treatment interruption.

All TB patients were registered, treated and managed according to the procedures of the National Tuberculosis Control Plan. They all started standardized treatment directly after registration. The standardized treatment regimen consists of two months of thrice-weekly isonizid, rifampicin, pyrazinamide and ethambutol followed by four months of thrice-weekly isoniazid and rifampicin. Patient supervision and follow-up with sputum smear tests during and at the end of treatment was conducted as per the NTP guidelines.

Information on chest radiograph results and sputum smear microscopy results at month 2-3, month 5 and month 6 was extracted from the TB patient medical record at the local CDC. Treatment outcomes were registered at the end of treatment according to the WHO definitions [2].

Questionnaires were pre-tested, and modified according to the lessons learned in the pre-test. Directly after the interview the filled in questionnaires were checked for completeness and consistency. The researchers re-interviewed a random sample of 10% of the included individuals and cross checked data with other data sources. Rechecking showed satisfactory data quality.

### Operational definitions

We used the definitions of the WHO for new smear-positive TB patient and treatment outcome [2]. Successful treatment includes cure and treatment completion, and unsuccessful treatment includes failure, death, and default. We also used the WHO definition for reporting of smear microscopy, where the reported result indicates the number of acid-fast bacilli observed. A result of at least 2 relates to at least 1 acid-fast bacillus observed per microscopy field [14].

Annual income was categorized into three groups of equal size: low income group <3000 Yuan (RMB); medium income group 3000-7000 Yuan (RMB); and high income group >7000 Yuan (RMB). One Yuan equalled about 0.13 USD in 2007.

According to China's TB Control Program (NTP) guidelines, anti-TB drugs should be taken every other morning (on even dates) before breakfast. Patients taking drugs at other times or intervals were considered as taking drugs not according to the guidelines.

Interruption of treatment means that patients missed at least one dose during treatment. Interruption of treatment was categorized as: (1) interruption of taking drugs during intensive phase, or (2) interruption of taking drugs during continuation phase, or (3) interruption of taking drugs during both phases.

### Data management and analysis

Individual patient data were double entered by different persons in Epi data version 3.0 (Epidata Association, Denmark, 2006), and differences were checked. Patient data were analyzed using R software version 2.7.1 (R, New Zealand, 2008). A simple descriptive analysis was performed using percentages, means, medians, and standard deviations (SD). For the univariate analyses χ^2 ^tests or Fisher's exact tests were used to compare the distribution of categorical variables between groups. The t-test was used to compare the distribution of age between participants and non-participants registered and treated by the TB dispensaries at the local CDCs.

Logistic regression analysis was conducted to identify risk factors for non-cure. Variables yielding a P-value ≤ 0.2 in the univariate analysis were included in the multivariate analysis, and the model was refined by backward elimination guided by the change in log likelihood of successive models with a significance level of 0.05.

### Ethical considerations

The principles of the Helsinki Declaration were taken into account. Written, informed consent was obtained from all study subjects. The study was approved by the medical ethical committee of the Chinese TB Control Association.

## Results

Of the 905 eligible TB patients registered between January and June 2007 in the 26 study counties, 59 (6.5%) refused to participate, leaving 846 patients. Of the 846 patients interviewed before start of treatment, 833 were also interviewed during the fifth month of treatment. Eight could not be interviewed again as they had died, and five had defaulted and could not be traced. Of the TB patients who refused to participate, 35 (59.3%) were male versus 576 (68.1%) of the participating TB patients (p = 0.28). Non participating patients were slightly older than participating patients (median age (SD), 39 (15.3) vs. 37 (15.7), p = 0.11). Of the patients that refused to participate, 9 (15.0%) had an unsuccessful treatment outcome.

The treatment success rate for the included smear positive pulmonary patients was 94%; 792 (93.6%) were cured and 5 (0.6%) completed treatment. Out of the 49 patients with unsuccessful treatment outcome, 17 (2.0%) failed treatment, 13 (1.5%) stopped prematurely due to side effects, 10 (1.2%) defaulted, and 9 (1.1%) patients died. Those who completed treatment without bacteriological confirmation of cure were deleted from the analyses as it was not clear whether they were actually cured, leaving 841 patients for the study. Including these 5 patients in the group with successful treatment outcome did not affect the results significantly (data not shown).

Table 1 shows the characteristics of the participants. Most patients were male (68.4%), under 30 years of age (33.9.%), had no formal education beyond primary school (62.6%), and had medical insurance (81.8%). Seventhy-one percent of patients delayed seeking health care for TB symptoms for more than 30 days,. Almost all (98.0%) had a sputum smear examination result after two months of treatment, and 11.7% of those were still sputum smear positive. Treatment was not observed for 27.7% of patients while for 49.3% of patients, treatment was observed by family members only. In 10.1% of cases, treatment was partly observed by health workers and partly by family members. Almost one in five patients (18.4%) indicated to have interrupted treatment. During the fifth month of treatment, 9.0% of patients were unaware that during the treatment period he/she should go the CDC three times for medical follow-up including sputum smear examination; 38.4% did not know the correct treatment duration, and 74.7% did not perceive direct observation of treatment as necessary. A large proportion of those who reported to be unaware of the need for follow-up had interrupted treatment (87% versus 22%, p < 0.001). Within this subgroup that had interrupted treatment and reported to be unaware of the need for follow-up, 52% had missed up to seven doses in a row and 48% had missed more than seven doses consecutively.

**Table 1:** Patient and treatment characteristics associated with non-cure in Yunnan province, China (N = 841)

*Characteristic*	*N(%)*	*non-cure (%)*	*RR*	*95% CI*	*p-value *
**Sex**					*0.27*
Male	575 (68.4)	37 (6.4)	1		
Female	266 (31.6)	12 (4.5)	0.69	0.35 - 1.3	
**Age (years)**					*0.66*
<30	285 (33.9)	18 (6.3)	1		
≥30	556 (66.1)	31 (5.6)	0.88	0.48 - 1.6	
Education level**					*0.01*
No formal education or primary school only	520 (62.6)	38 (7.3)	1		
Secondary school or higher	311 (37.4)	10 (3.2)	0.42	0.21 - 0.86	
**Medical insurance **					*0.02*
No	153 (18.2)	15 (9.8)	1		
Yes	688 (81.8)	34 (4.9)	0.48	0.32 - 0.90	
Income*,**					*0.11*
Low	314 (37.3)	21 (6.7)	1		
Middle	239 (28.4)	18 (7.5)	1.1	0.59 - 2.2	
High	288 (34.3)	10 (3.5)	0.50	0.23 - 1.12	
**Co-morbidity **					*0.06*
No	755 (89.8)	40 (5.3)	1		
Yes	86 (10.2)	9 (11.6)	2.1	0.98 - 4.5	
**Patient delay (days) **					*0.02*
<30	243 (28.9)	7 (2.9)	1		
≥30	598 (71.1)	42 (7.5)	2.5	1.1 - 5.8	
**Directly observed treatment**					*0.01*
Yes, by health care staff	108 (12.8)	4 (3.7)	1		
Yes, shared by health care staff and family member	85 (10.1)	9 (10.6)	3.1	0.9 - 10.4	
Yes, by family member	415 (49.3)	15 (1.8)	0.98	0.32 - 3.0	
No	233 (27.7)	21 (9.0)	2.6	0.86 - 7.7	
**Sputum smear status before treatment **					*0.03*
≤2+	503 (59.8)	22 (4.4)	1		
>2+	338 (40.2)	27 (8.0)	1.9	1.1 - 3.4	
**Sputum smear result after 2 months of treatment **					*0.01*
Negative	726 (86.3)	21 (2.9)	1		
Positive	98 (11.7)	11 (11.1)	4.2	2.0 - 9.1	
Without test	17 (2.0)	17 (100)	34.8	24.7 - 48.6#	
Adverse events**					*0.06*
No	539 (65.8)	22 (4.1)	1		
Yes, did not require hospitalization	262 (32.0)	10 (3.8)	0.93	0.44 - 2.0	
Yes, required hospitalization	18 (2.2)	3 (16.7)	4.7	1.3 - 17.4	
**Interruption of treatment**					*0.17*
No	686 (81.6)	39 (5.7)	1		
Yes, during intensive phase	28 (3.3)	3 (10.7)	2.0	0.58 - 6.9	
Yes, during continued phase	96 (11.4)	3 (3.1)	0.54	0.16 - 1.8	
Yes, during intensive and continuation phase	31 (3.7)	4 (12.9)	2.5	0.82 - 7.4	
Maximum number of missed doses in a row**					*0.09*
0	682 (81.6)	35 (5.1)	1		
1-7	102(12.2)	3 (2.9)	0.56	0.17 - 1.9	
>7	52 (6.2)	6 (11.5)	2.4	0.97 - 6.0	
**Taking drugs according to guideline **					*< 0.001*
Yes	638 (75.9)	19 (3.0)	1		
No	203 (24.1)	30 (14.8)	5.7	3.1 - 10.3	
Patient knows that during treatment he/she should go to CDC for medical check-ups**					*< 0.001*
Yes	752 (91.0)	22 (2.9)	1		
No	74 (9.0)	14 (18.9)	7.7	3.8 - 15.9	
**Patient knows the duration of TB treatment **					*0.001*
Yes	518 (61.6)	19 (3.7)	1		
No	323 (38.4)	30 (9.3)	2.7	1.5 - 4.9	
Patient agrees with the need for treatment observation**					*0.06*
Yes	209 (25.3)	4 (1.9)	1		
No	616 (74.7)	31 (5.0)	2.7	0.95 - 7.8	
Family attitude**					*0.07*
More supportive than usual	339 (40.3)	9 (2.7)	1		
No change or less supportive than usual	502 (59.7)	40 (8.0)	2.1	0.95 - 4.5	

RR = Relative Risk, 95%CI = 95% confidence interval

* based on tertiles

# As there is one cell with a zero, we cannot estimate RR directly. We added 0.5 to each cell to estimate the RR.

** numbers do not add up to 841 due to missing values

In univariate analysis, patient characteristics significantly associated with non-cure were having no medical insurance, patient delay in seeking care for TB symptoms >30 days, and high initial sputum acid fast bacilli load (>2+) (Table 1). Treatment-related characteristics significantly associated with non-cure in univariate analysis were still having a positive sputum smear after two months of treatment, not having a DOT supervisor, and not taking drugs according to the NTP guideline. Also, patients who indicated that observation of treatment was not necessary, patients who were unaware of the need to go at least three times to the local CDC during treatment, and patients being unaware of the correct treatment duration were at increased risk of non-cure outcome.

Patient and treatment characteristics independently associated with non-cure were having no medical insurance, a low income, patient delay >30 days, a positive 2-month smear test result, being unaware of the need to go to local CDC for check-ups during treatment, and refusal of direct observation of taking drugs. Patients who reported to have interrupted treatment for less than two weeks less often were cured than patients who reported never to have missed a dose (Table 2).

**Table 2:** Independent risk factors for non-cure in Yunnan province, China, identified by multivariate logistic regression analysis (N = 813^#^ )

*Characteristic*	*OR (95% CI)*	*p-value*
**Medical insurance **		*0.002*
yes	1	
no	4.7 (1.8 - 12.3)	
Income*		*0.05*
low	1	
middle	0.45 (0.16 - 1.3)	
high	0.22 (0.06 - 0.82)	
**Patient delay (days) **		*0.25*
<30	1	
≥30	6.3 (1.3 - 32.0)	
**Sputum smear status before treatment**		*0.05*
≤2+	1	
>2+	2.7 (1.0 - 7.1)	
**Sputum smear result after 2 months of treatment **		*<0.001*
negative	1	
positive	6.0 (2.3 - 15.9)	
**Maximum number of missed doses in a row**		*0.09*
0	1	
1-7	0.06 (0.04 - 0.74)	
>7	0.52 (0.08 - 3.6)	
**Taking drugs according to guideline **		*0.03*
yes	1	
no	2.8 (1.1 - 7.1)	
**Patient knows that during treatment he/she should go to CDC for medical check-ups **		*0.01*
yes	1	
no	9.9 (1.7 - 57.6)	
**Patient agrees with the need for treatment observation **		*0.05*
yes	1	
no	4.4 (0.98 - 20.1)	

OR = Odds Ratio, 95%CI = 95% confidence interval

* based on tertiles

**# **841 patients in the study, but the questionnaire of 28 patients was not complete, so only 813 patients could be included in the multivariate model

## Discussion

Many factors were observed to be related to non-cure in this study. Independent risk factors for non-cure of new smear-positive TB patients in the selected counties with a low cure rate in Yunnan province were: having no medical insurance; a low income, patient delay >30 days; a positive 2-month smear test result; being unaware of the need to go to local CDC for check-ups during treatment; and refusal of direct observation of drugs taking.

Almost one in ten patients (9.0%) were unaware of the need to go to local CDC for medical check-ups including sputum smear examinations. These patients had an eight times higher risk of non-cure than patients who knew that they should attend medical check-ups at the CDC. More than one in ten patients (11.7%) still had a positive sputum smear after two months of treatment, which is lower than the 18% observed in the registration data of Yunnan province in 2007 (unpublished data). Patients who were still sputum smear positive after two months had a six to seven times increased risk of non-cure. Other independent risk factors for non-cure were not having medical insurance, a low income, patient delay in health care seeking for TB symptoms >30 days, not taking drugs according to the guideline, and not seeing the need for observation of treatment. Patients who indicated that they never had missed a dose more often were not cured than patients who indicated to have missed a maximum of 1-7 doses in a row. This is in contradiction with results of previous studies [4,15-17]. In these studies treatment interruption was associated with an increased risk of non-cure. The contradiction may partly be explained by the fact that many patients who died or failed treatment had not missed any dose according to the information provided: 92% versus 95% among those cured. Furthermore, it may be that some patients that reported not to have missed a single dose might be afraid to admit that in fact they had missed some doses. Especially patients that did not respond well to treatment may be less likely to admit that they missed doses.

Patients with positive smears after 2 months of treatment may not react well to treatment due to an infection with drug resistant *Mycobacterium tuberculosis*, one of the major risk factors for non-cure. Drug susceptibility testing and treatment for multi-drug resistant tuberculosis were not implemented within the NTP in Yunnan province during the study, and no drug resistance survey has been conducted. We were unable to obtain information about the presence of drug resistance [18].

Patients with a DOT observer had a decreased risk of non-cure compared to patients without a DOT observer in univariate analysis, as has been observed in other studies [19-21]. Half of the patients had a family member as DOT observer and their outcomes were similar to the patients who had a health care worker as DOT observer. A recent study from Tanzania also observed similar treatment outcomes for sputum smear positive TB patients with community members, mostly family members, being DOT supervisors, compared to those with facility-based DOT [22].

Treatment was not observed at all for 28% of patients. DOT is the cornerstone of the DOTS strategy in China. In our data, lack of treatment observation was not an independent risk factor when adjusting for other factors influencing treatment outcome. This could be due to the fact that quality of DOT is not always sufficient, even if performed by health care workers like village doctors [12,23]. If it is not possible to increase the quality of DOT by village doctors, e.g. due to the high workload of village doctors, family members could provide DOT in China. A prerequisite is that they are trained well, and remain motivated and committed to the program.

Our study results support the results of previous reports that stated that delayed care seeking (RR = 2.7) and a high bacillary load at diagnosis (which may result from delayed care seeking) also increase the risk of non-cure [4,16,24-27]. Public awareness of presence of TB dispensaries and the availability of free TB diagnosis and treatment is low in China. In a recent survey 42% of the interviewees were aware of the existence of TB dispensaries and 45% knew about the free diagnosis and treatment policy for TB [28].

Patients without medical insurance more frequently were not cured. In China, sputum smear examinations, chest radiographs and anti-TB drugs are provided free of charge, but additional costs, e.g. treatment of side-effects are not [29,30]. Medical insurance covers part or all of these extra costs. The financial burden for those with no or partial insurance has been described to affect treatment adherence [10,31]. The financial burden is highest for those with a lower income. The 26 counties with a cure rate below 85% had a lower average gross domestic product per capita than the other counties in Yunnan province (11,980 versus 13,826 RMB). Also the number of health care workers per capita was lower (218 versus 244 per 100,000 inhabitants). These factors both may be an explanation for a lower overall cure rate.

Our study has a number of limitations. We included counties with a cure rate below 85% in 2005 in our study. During the study period in 2007, the average cure rate in these counties had increased to 94%. The lower number of non-cured patients limited the statistical power to detect determinants of unsuccessful treatment. Furthermore, a lower successful treatment outcome percentage was observed among non-participants than among participants (15% vs. 6%). Because the number of non-participants with unsuccessful treatment outcome was relatively small, we do not expect that non-participation in this study will have biased our findings to a relevant extent.

## Conclusions

Our study has provided us with useful insights on factors influencing non-cure in Yunnan province. Based on the results, we recommend that patient education by clinic doctors should be enhanced to improve the patients' understanding of their disease and its treatment and to improve compliance with treatment and follow up. More resources and effort are needed to educate the general public to enable them to act effectively as community-based treatment observers.

## List of abbreviations used

TB: tuberculosis; PTB: Pulmonary tuberculosis; NTP: China's TB Control Program; DOT: Observation of drug intake; DOTS: directly observed treatment short-course; CDC: Center for Disease Control and Prevention; SS+: Sputum smear positive; RR: relative risk; 95%CI: 95% Confidence interval.

## Competing interests

The authors declare that they have no competing interests.

## Authors' contributions

HJZ designed the study and wrote the manuscript. SH has been involved in monitoring of data-collection, data-analysis, and revision of the manuscript. XL, QYB and HJL supervised data collection and were involved in data-analysis. MJW has been involved in study design and revision of the manuscript. All authors have read the manuscript repeatedly and approved its final version.

## Pre-publication history

The pre-publication history for this paper can be accessed here:

http://www.biomedcentral.com/1472-6963/11/97/prepub
